# Efficiency of Household Reactive Case Detection for Malaria in Rural Southern Zambia: Simulations Based on Cross-Sectional Surveys from Two Epidemiological Settings

**DOI:** 10.1371/journal.pone.0070972

**Published:** 2013-08-06

**Authors:** Kelly M. Searle, Timothy Shields, Harry Hamapumbu, Tamaki Kobayashi, Sungano Mharakurwa, Philip E. Thuma, David L. Smith, Gregory Glass, William J. Moss

**Affiliations:** 1 Department of Epidemiology, Bloomberg School of Public Health, Johns Hopkins University, Baltimore, Maryland, United States of America; 2 W. Harry Feinstone Department of Molecular Microbiology and Immunology, Bloomberg School of Public Health, Johns Hopkins University, Baltimore, Maryland, United States of America; 3 Macha Research Trust, Choma, Zambia; Tulane University School of Public Health and Tropical Medicine, United States of America

## Abstract

**Background:**

Case detection and treatment are critical to malaria control and elimination as infected individuals who do not seek medical care can serve as persistent reservoirs for transmission.

**Methods:**

Household malaria surveys were conducted in two study areas within Southern Province, Zambia in 2007 and 2008. Cross-sectional surveys were conducted approximately five times throughout the year in each of the two study areas. During study visits, adults and caretakers of children were administered a questionnaire and a blood sample was obtained for a rapid diagnostic test (RDT) for malaria. These data were used to estimate the proportions of individuals with malaria potentially identified through passive case detection at health care facilities and those potentially identified through reactive case finding. Simulations were performed to extrapolate data from sampled to non-sampled households. Radii of increasing size surrounding households with an index case were examined to determine the proportion of households with an infected individual that would be identified through reactive case detection.

**Results:**

In the 2007 high transmission setting, with a parasite prevalence of 23%, screening neighboring households within 500 meters of an index case could have identified 89% of all households with an RDT positive resident and 90% of all RDT positive individuals. In the 2008 low transmission setting, with a parasite prevalence of 8%, screening neighboring households within 500 meters of a household with an index case could have identified 77% of all households with an RDT positive resident and 76% of all RDT positive individuals.

**Conclusions:**

Testing and treating individuals residing within a defined radius from an index case has the potential to be an effective strategy to identify and treat a large proportion of infected individuals who do not seek medical care, although the efficiency of this strategy is likely to decrease with declining parasite prevalence.

## Introduction

In the past decade, international support and funding for malaria control increased dramatically and targets were set to reduce the burden of malaria by 75% by 2015 and eliminate malaria in 8–10 countries by 2015 [1]. This renewed commitment to malaria elimination has been made possible with increased coverage of four key interventions: long-lasting insecticide-treated nets (ITNs), indoor residual spraying (IRS), case identification with rapid diagnostic tests (RDT) and treatment with artemisinin-combination therapy (ACT), and intermittent preventive treatment for pregnant women and infants. Programs that achieved high coverage with these interventions showed dramatic decreases in the number of malaria cases, hospital admissions and deaths [1]–[4] and 11 African countries demonstrated large (>50%) and sustained decreases in the burden of malaria [1].

Case detection and treatment are critical to malaria elimination as infectious individuals serve as reservoirs for transmission [5]. Several case detection strategies have been developed and implemented. Passive case detection, involving identification of symptomatic patients seeking care at health facilities based on RDT or microscopy, requires the least resources. This strategy, however, does not identify asymptomatic (those with no symptoms), minimally symptomatic (those with mild symptoms or the perception that symptoms do not require medical treatment), or symptomatic, infected individuals who do not seek medical care, as these individuals do not present to health care facilities. The proportion of all infected persons who are asymptomatic, minimally symptomatic or do not seek medical care can be substantial and as high as 96% [6]–[8], suggesting that a majority of infectious cases could be missed with passive case detection.

Reactive case detection [9] extends this strategy based on the assumption that malaria cases are spatially clustered and that cases identified at health centers (index cases) represent foci of infection within households and surrounding neighborhoods. With reactive case detection, residents of households of index cases, and possibly of neighboring households, are screened using RDT and offered treatment if infected. In a study of reactive case detection in rural southern Zambia, the prevalence of malaria was found to be significantly higher among residents of households of index cases than among residents of randomly selected households in the study area [10]. Importantly, both passive and reactive case detection strategies based on standard diagnostic tests (RDT and microscopy) fail to identify individuals with low-level parasitemia below the limits of detection of these tests.

Little data exist, however, on the appropriate radius from the index household that should be screened with reactive case detection using RDT, and the efficiency and cost-effectiveness of this radius likely varies in different epidemiological settings. Using serial cross-sectional household surveys and model simulations in two settings with different levels of malaria transmission in southern Zambia, we sought to quantify the efficiency of screening individuals within households and the neighbors of index cases who present for treatment at health care facilities, and to estimate the radii necessary to achieve different levels of treatment coverage.

## Methods

### Study Site

**Figure 1:**
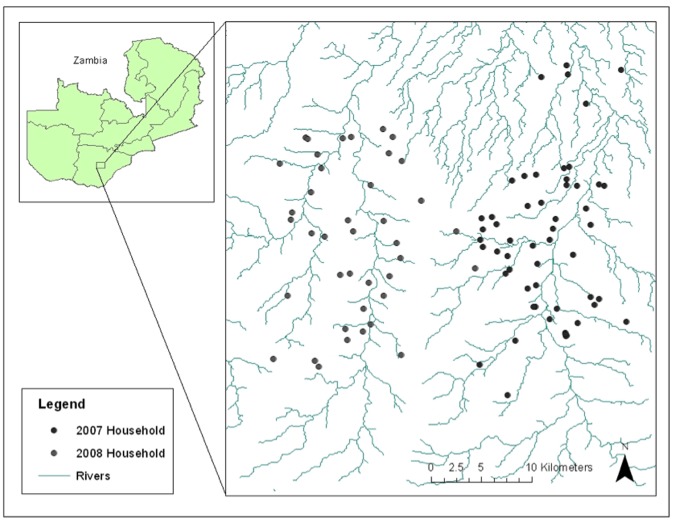
Map of the 2007 and 2008 study sites in Choma District, Southern Province, Zambia.

The study was conducted in two epidemiological settings within the catchment area of Macha Hospital in Choma District, Southern Province, Zambia between April 2007 and December 2008. Households sampled in 2008 were selected from a different geographic area than those sampled in 2007 (Figure 1). Macha Hospital is approximately 70-kilometers from the town of Choma and lies on a plateau 1,100-meters above sea level. The single rainy season lasts from December through April, followed by a cool season from April until August, and a hot dry season through November. The primary malaria vector in this region is *Anopheles arabiensis*, and transmission peaks during the rainy season (December-April) [11]. The catchment area is populated by villagers living in small, scattered homesteads. Southern Province, Zambia was reported to have hyperendemic *P. falciparum* transmission [12]. However, the prevalence of malaria has declined over the past decade [13]. ACTs were introduced as first-line anti-malarial therapy in Zambia in 2002 [14] and into the study area in 2004, and insecticide treated bed nets (ITNs) were widely distributed in the study area in 2007 [14].

### Study Population

The development of the sampling frame and enumeration of households were reported elsewhere [14]. Briefly, satellite images were used to construct a sampling frame from which households were selected by simple random sampling for enrollment into prospective longitudinal and cross-sectional surveys of malaria parasitaemia. Households enrolled in the longitudinal cohort were repeatedly surveyed every two months, whereas households enrolled in the cross-sectional cohort were surveyed once. The household survey was conducted from April through December in 2007 and from February through December in 2008 [14]. This analysis was restricted to households enrolled in the cross-sectional surveys and the first study visit of households enrolled in the longitudinal surveys.

The study was approved by the University of Zambia Research Ethics Committee and the Institutional Review Board at the Johns Hopkins Bloomberg School of Public Health. Informed consent was obtained from all participants. During each study visit, a questionnaire was administered to consenting participants over 18 years of age and to the guardians of participants younger than 18 years of age. Data collected included demographic information, current signs and symptoms of malaria, history of recent malaria and antimalarial treatment, reported health seeking behavior, knowledge of malaria transmission and prevention, and the use of ITNs. Participant’s temperature was measured using a Braun Thermoscan® ear thermometer. A blood sample was collected by finger prick for malaria rapid diagnostic testing (RDT). The RDT (ICT Diagnostics, Cape Town, South Africa) detected *P. falciparum* histidine-rich protein 2 and was shown to detect 82% of test samples with wild-type *P. falciparum* at a concentration of 200 parasites/µL and 98% of test samples with a concentration of 2000 parasites/µL, with false positives in 0.6% of negative samples [15]. Participants who were RDT positive were offered treatment with artemether-lumefantrine (Coartem**®**).

### Spatial Risk Map

A spatial risk map was previously developed using ecological and survey data [14]. Logistic regression was used to identify environmental factors associated with the odds of a household having an RDT positive resident. Each household in the study area was assigned a malaria risk according to its location on the spatial risk map ranging from. 065 to. 797, referred to as the ecological risk.

### Sample Survey Data

Data from 2007 and 2008 were analyzed to compare characteristics under the different transmission settings represented by each year. For each year, differences between RDT positive and RDT negative individuals were compared using Fisher’s exact test for dichotomous variables and two-sample t test for continuous variables. The Wilcoxon-ranksum test was used to compare mean ages between households.

### Generation of Passively Detected Index Cases

Individuals were classified as likely to be passively detected (index cases) if they were RDT positive, had malaria specific symptoms and displayed care-seeking behavior. Care seeking behavior was determined if the individual reported visiting a health post or clinic for their most recent febrile illness. Malaria specific symptoms consisted of having a fever with either a headache or chills in the prior two weeks. An alternative algorithm was developed for individuals receiving antimalarial medication at the time of the survey. Individuals currently taking antimalarials from a health care facility, and who thus displayed care-seeking behavior, were classified as likely to be passively detected index cases.

RDT positive individuals likely to be detected and missed through passive case detection based on the algorithm were compared based on care seeking behavior, symptoms and ecological risk using Fisher’s exact test and two-sample t test.

### Classification of Households and Individuals Detected through Reactive case Detection

All households with one or more RDT positive resident were classified as positive households. Positive households were further classified as “identified” or “missed” based on whether or not at least one RDT positive resident was likely to be passively detected (i.e. was classified as seeking care for malaria-like illness). Individuals likely to be detected through reactive case detection were those who were RDT positive but were asymptomatic or minimally symptomatic, did not display care seeking behavior, or both, but resided in a household likely to be identified.

Positive households were compared on the basis of being identified or missed. The variables used for analysis included: mean age of household residents, number of residents in the household, number of RDT positive residents in the household, number of symptomatic and asymptomatic RDT positive residents in the household, and the household ecological risk.

### Spatial Analysis of Sample Survey Data

Positive households were mapped using ArcGIS version 10.0 (Environmental Systems Research Institute [ESRI], Redlands, California). The identified and missed households were added as data layers geo-referenced to Universal Transverse Mercator (UTM), Southern Hemisphere, Zone 35, WGS1984. Identified and missed households were uniquely coded and distances between identified and missed households were determined. These distances were used to determine radii around identified households that would potentially need to be traversed to identify missed households.

### Population Level Simulation

Simulations were performed using predictive models to extrapolate from sampled to non-sampled households based on household level data from those surveyed in 2007 and 2008. Individual and household survey data were selected from the dataset to create a household level dataset with covariates of interest for the development of predictive models. From this aggregated household level dataset, predictive models were determined for each covariate of interest to locate houses that would potentially be identified, according to the passive case detection and household identification algorithms, in order to fit the optimal chained equations to be used in the simulation. Dichotomous covariates of interest were predicted using logistic regression and continuous covariates of interest were predicted using linear regression. Each of the following variables was predicted at the household level: RDT status, antimalarial treatment status, number of RDT positive residents, at least one symptomatic resident, at least one care seeking resident, at least one symptomatic and care seeking RDT positive resident, and residents treated for malaria who sought care. For the predictive models, geographic coordinates, ecological risk, mean age of household residents, and number of household residents were used as initial predictive covariates.

Logistic regression models were evaluated using the Hosmer & Lemeshow goodness-of-fit test and the area under the receiver-operating curve (AUC). Linear regression models were evaluated using the R^2^. The predictive models were initially built using the 2007 household level data. The AUC measurements for all dichotomous models were greater than 0.70 and p-values for the Hosmer & Lemeshow goodness-of-fit test were greater than 0.05 (Table S1 in File S1). The R^2^ values for continuous models were greater than 0.50. The models were validated using the 2008 household data to ensure that the same model was fit under both transmission settings. Using the 2008 household data, the AUC measurements for all dichotomous models were greater than 0.65 and p-values for the Hosmer & Lemeshow goodness-of-fit test were greater than 0.05 (Table S2 in File S1). The R^2^ values for continuous models were greater than 0.50. Households with ecological risk of less than 0.196 were not included in the simulation and were assumed to be negative (i.e. no RDT positive residents).

The simulation was performed using a multiple imputation by chained equations (MICE) method in STATA version 12.0 (StataCorps, College Station, TX), also referred to as fully conditional specification or sequential regression multivariate imputation [16]–[18]. In this analysis, all sampled household values were observed while non-sampled households had full data only for geographic coordinates and ecological risk of malaria. All other values for non-sampled households were missing. With MICE, initially all missing values are temporarily filled by a simple random sample of the observed values [16]. The first variable imputed in the chain is regressed on the variables specified by the model to predict that variable as well as the observed values for this variable. The subsequent variables imputed in the chain are regressed on the variables specified by their prediction model as well as their observed values, with the addition of variables previously imputed in the chain that are in their prediction model. MICE enabled the incorporation of multiple predictive covariates to simulate the population represented by the sampled households and allowed the use of outcome values imputed for a household to be used in the prediction of outcomes imputed in each subsequent chain.

All non-sampled households had covariates for ecological risk and longitude and latitude coordinates (i.e. X and Y coordinates). Numbers of persons per household and household mean age for non-sampled households were predicted first in the chain using predicted mean matching based on covariates from the survey sample data. Household RDT status (having at least one RDT positive individual in a household) and household antimalarial medication status (having at least one person receiving antimalarial medication in the household) were predicted next in the chain from the ecological risk, household spatial coordinates, persons per household (household sample level and imputed), and household mean age (household sample level and imputed).

The number of RDT positives per household was imputed next in the chain from the ecological risk, household coordinates, persons per household (household sample level and imputed), household mean age (household sample level and imputed), and household RDT status (household sample level and imputed). Restrictions were placed on this predicted outcome to ensure that the number of RDT positive residents per household did not exceed the number of persons per household and, if the household was predicted to be positive, the number of RDT positive residents was at least one. Additionally, if the household was predicted to be RDT negative, no RDT positive residents resided in the household.

RDT positive residents with symptoms and care seeking behavior were imputed next in the chain from the ecological risk, household coordinates, persons per household (household sample level and imputed), household mean age (household sample level and imputed), household RDT status (household sample level and imputed), number of positives per household (household sample level and imputed) and household antimalarial medication status (household sample level and imputed). A person who received antimalarial medication in a household and visited a healthcare facility to obtain the medication was imputed last in the chain from the ecological risk, household coordinates, persons per household (household sample level and imputed), household mean age (household sample level and imputed), household RDT status (household sample level and imputed), and antimalarial medication status of the household (household sample level and imputed).

The simulated data were assessed to ensure that the simulated household population (persons per household), simulated mean household age and simulated household level malaria prevalence did not differ significantly from the sampled data. Since only household level data were used in the predictive models, if a simulated RDT positive household was classified as likely to be identified, all simulated RDT positive residents of that household also were classified as likely to be identified.

The prediction models used to perform the imputation were evaluated with the simulated data for each year to ensure that the models fit the simulated data. The same methods for evaluating the models in the sampled data were used to evaluate the models in the simulated data (Tables S3 and S4 in File S1).

### Spatial Analysis of Population Level Simulated Data

Simulated RDT positive households were plotted on the map of the study area and differentiated as identified or missed. The identified and missed households were added as data layers using the projected Universal Transverse Mercator (UTM), 1983 Southern Hemisphere, Zone 35 coordinate system. Distance-based buffers were set surrounding identified households (index households). These buffers represented varying distances surrounding identified households that would be screened for malaria to detect and treat RDT positive individuals. Buffers of different distances were evaluated to determine the buffer size needed to identify maximum proportions of missed positive households under each transmission setting (2007 and 2008). In addition to using the distances provided from the sample data, buffer distances ranging from 500 to 3,000 meters surrounding an index household were evaluated. The buffers were dissolved to ensure that a household could only be counted once in the event that a missed household was located within the buffer of more than one identified household. Each buffer layer was then spatially joined to the missed household data layer. The sum of all missed households (as well as residents likely to be RDT positive in missed households) within each buffer layer, the proportions of missed RDT positive households and residents within each buffer of identified RDT positive households (relative to all RDT positive missed household), and the proportions of all RDT positive households within each buffer were calculated.

In addition, negative households from the simulation and those assumed to be negative by having an ecological risk less than 0.196 were added as new data layers to the map. The sum of all negative households within each buffer layer, and the proportions of negative households that potentially would be screened within each buffer (relative to all households screened in each buffer) were calculated. These proportions were then compared to proportions of positive households screened within each buffer to determine the impact of reactive case finding in each transmission setting.

Statistical analyses were performed using STATA 12.0 (StataCorps, College Station, TX). Spatial analyses were performed using ArcGIS 10.0 (Environmental Systems Research Institute [ESRI], Redlands, California).

## Results

### Characteristics of Sampled Households

The 2007 study area represented a setting of moderate transmission with a parasite prevalence of 23% by RDT, whereas the 2008 data represented a setting of low transmission (recently transitioned from moderate transmission) with a parasite prevalence of 8% by RDT. Two demographic characteristics differed significantly across the two study sites and years, care seeking behavior (43% in 2007 vs. 56% in 2008; p = .001) and reported malaria symptoms (37% in 2007 vs. 24% in 2008; p<.001).

### Households Sampled in the 2007 Study Area

In 2007, RDT positive individuals were younger than RDT negative individuals (mean age 13.4 years vs. 23.4 years, p<.001) and were more likely to report symptoms consistent with malaria during the previous two weeks than RDT negative individuals (53.0% vs. 32.9%, p = .004). RDT positive and negative participants did not differ significantly on other demographic characteristics analyzed. Only 13 of 66 (19.7%) RDT positive participants would likely have been identified in 2007 using passive case detection using the algorithm. Of the remaining RDT positive participants, 11 (16.7%) were symptomatic with no care seeking behavior, 13 (19.7%) were asymptomatic with care seeking behavior, and 29 (43.9%) were asymptomatic with no care seeking behavior. With reactive case detection of household members residing with an index case, an additional 20 RDT positive malaria cases would likely have been detected, resulting in identification of half (33 of 66) of all RDT positive individuals among the sampled households. Of the RDT positive persons likely to have been identified, 73% were symptomatic. In contrast, only 33.3% of the RDT positive persons missed were symptomatic. No significant differences were observed for care seeking behavior or ecological risk of RDT positive persons identified and missed in 2007.

For all individuals residing in sampled households, those identified or residing in an identified household were more likely to be RDT positive (34% vs.17.6% p = .003), have malaria specific symptoms (58.8% vs. 26.6% p<.001) and have care seeking behavior (55.7% vs. 36.7% p = .002) than those not identified or residing in missed households. There were no significant differences observed for ecological risk. Thirty-five of 48 households (73%) had either an RDT positive individual or an individual receiving antimalarial drugs from a health care facility. Of these households, 41% would likely have been identified using the algorithm through passive case detection.

### Households Sampled in the 2008 Study Area

In 2008, RDT positive and negative participants differed in numbers of persons per household, with RDT positive persons residing in larger households (82.3% in households with 5 or more persons per household vs. 60.9% in households with less than 5 persons per household; p = .03). RDT positive and negative individuals did not differ significantly by other demographic characteristics. Four of 34 (12%) RDT positive cases would likely have been identified through passive case detection using the algorithm. Nine additional cases would likely have been identified through reactive case detection within the household, resulting in detection of 13 of 34 (38%) of all RDT positive individuals within the study area. Of the RDT positive individuals, there were no differences in symptoms, care seeking behavior or ecological risk, between those identified and missed using reactive case finding.

For individuals residing in sampled households, those identified or residing in an identified household were more likely to be RDT positive (22.81% vs.6.03%, p<.001) and to reside in an area of slightly lower ecological risk (0.310 vs. 0.388, p<.001) than those not identified or residing in missed households. There were no differences between symptoms and care seeking behavior among individuals identified and missed. Twenty-two of 75 households (29%) had either an RDT positive individual or a person taking antimalarial medication received from a health care facility. Of these households, 41% would likely have been identified using the algorithm, through passive case finding.

### Simulated Data from Households Surveyed in 2007

Extrapolation from the households surveyed in 2007 to the non-sampled households resulted in data estimated for 7,980 households with 47,058 individual residents. Household level characteristics of the simulated households did not differ significantly from the sampled households with the exception of the household level of care seeking behavior (i.e. an individual in the house displays care seeking behavior): 70.8% in the sampled households and 86.82% (p = .004) in the non-sampled households (Table 1).

**Table 1:** Characteristics of sampled and simulated households: 2007 and 2008.

	2007			2008		
	SampledHouseholds	SimulatedHouseholds	p-value	SampledHouseholds	SimulatedHouseholds	p-value
**Number of households**	48	7,980		75	7,961	
**Number of individuals screened**	284	47,058		403	42,620	
**Residents per household (mean, SD)**	5.93 (3.13)	5.90 (3.07)	.927	5.37 (2.69)	5.35 (2.65)	.949
**Mean age (mean, SD)**	25.26 (15.77)	25.04 (15.37)	.942	26.74 (16.32)	26.76 (16.13)	.975
**Households with an RDT positive individual (%)**	66.7	74.4	.245	24.0	16.8	.119
**Households with an individual taking antimalarials (%)**	10.4	21.1	.076	9.3	22.1	.007
**Households with an individual with care seeking behavior (%)**	70.8	86.8	.004	85.3	82.4	.647
**Households with an individual with malaria-like symptoms (%)**	75.0	70.0	.529	64.0	70.0	.258
**Households with an individual with malaria-like symptoms** **and care seeking behavior (%)**	37.5	31.1	.349	34.7	34.1	.903
**Households with an individual taking antimalarials with** **care seeking behavior (%)**	22.9	24.7	.868	6.7	4.5	.067
**Total households identified through reactive case detection (%)**	34.3	42.5	.393	40.9	49.9	.521

The simulation resulted in 5942 of 7,980 (74.4%) households having an RDT positive resident, with 2,397 (40.3%) of these households likely to have been identified through passive case detection (i.e. index households with a symptomatic, RDT positive individual who would seek care), and 3,545 (59.7%) households likely not to have been identified through passive case detection because the infected individuals were asymptomatic, did not seek care, or both (Table 1).

### Spatial Analysis of Simulated Data from the 2007 Survey Sample

**Figure 2:**
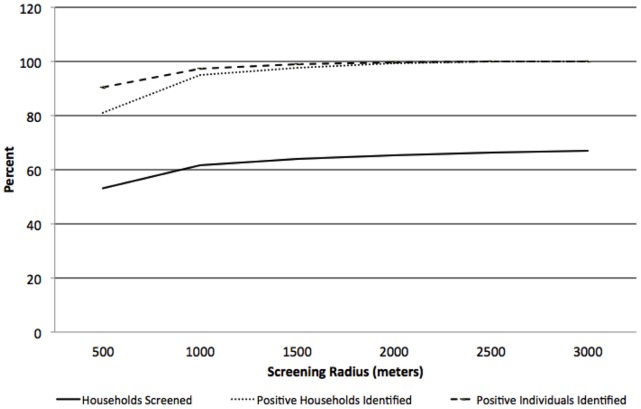
Percentage of RDT positive households identified, RDT positive individuals identified and total households screened by screening radii surrounding index households: 2007.

**Figure 3:**
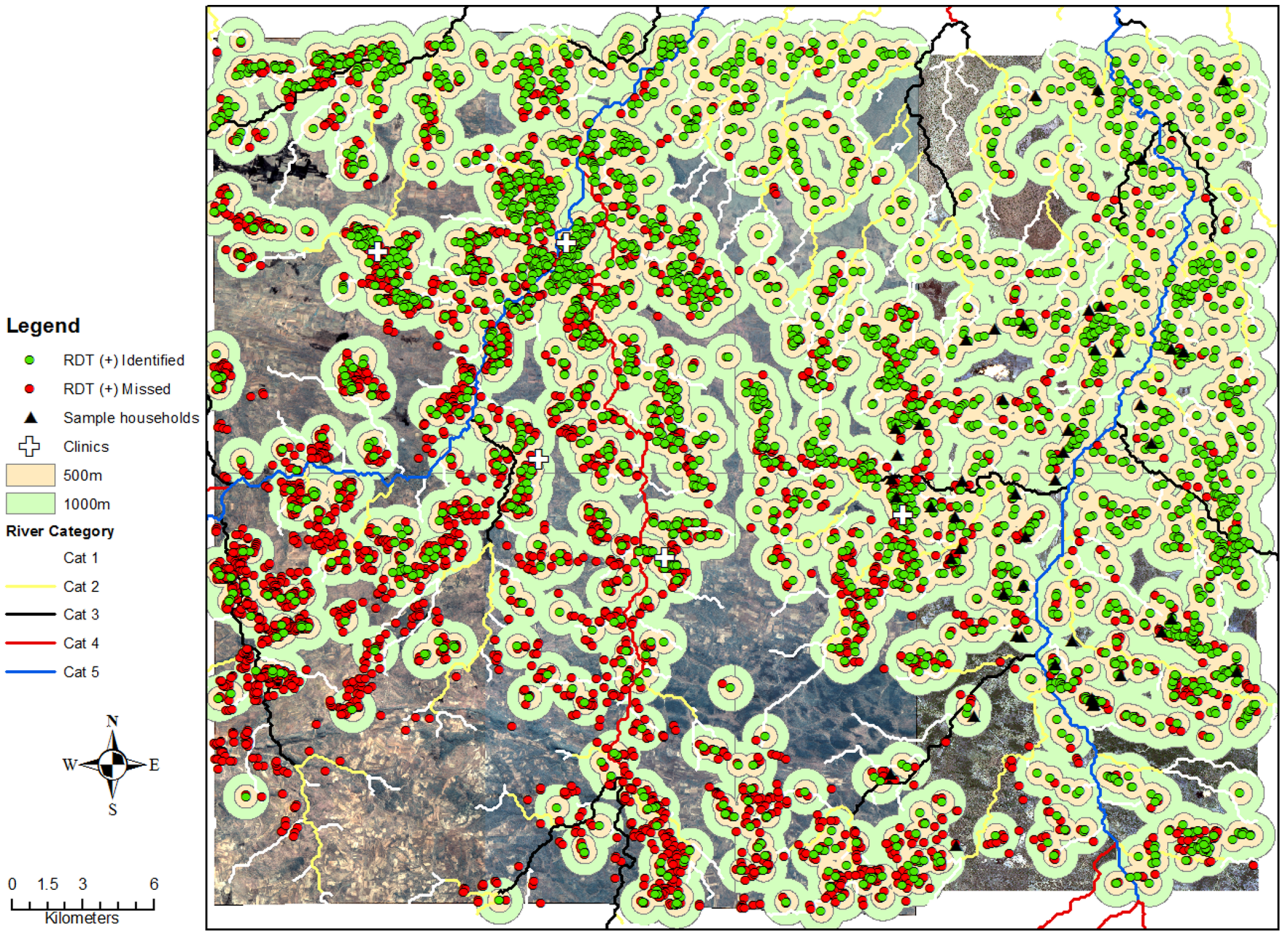
Map of screening radii surrounding RDT positive identified and missed households: 2007.

Of the non-identified households, 2,873 (81%) were located within a 500 meter radius of an index household and 3,362 (94.8%) were located within a one kilometer radius of an index household. When the radius surrounding index households was expanded to two kilometers, 3,519 (99.3%) of the non-identified households were within this range. All non-identified households were within a three kilometer radius of an identified household (Table 2). Testing and treating individuals residing within 500 meters of an index household identified 81% of households missed through passive case finding and 79% of all RDT positive individuals who would not have been identified and treated in a health care facility (Table 2). Of all households in the 500 meter radius, 62% were positive households, with a total of 53% of all households screened (Table 2, Figure 2). When combined with the RDT positive index households and residents, this strategy of screening all households within 500 meters of an index household would result in identifying 89% of all households with an RDT positive resident and 90% of all RDT positive individuals. If reactive case detection were increased from 500 meters to one kilometer from all index households, 95% of all households with an RDT positive resident and 94% of all RDT positive individuals would be identified, with 62% of all households screened (Table 2, Figures 2 and 3).

**Table 2:** Proportions of positive and negative households, missed households, missed individuals, total households, and total individuals identified at various screening radii: 2007 and 2008.

Buffer (m)	Positivehouseholds	Negativehouseholds	Total householdsscreened (%)	Missed positivehouseholds identifiedthrough reactive casedetection (%)	Total positivehouseholdsidentified throughreactive casedetection (%)	Total positiveindividuals	Missed individualsidentified throughreactive casedetection (%)	Total positiveindividualsidentified throughreactive casedetection (%)
**2007**								
500	2873	1778	53.2	81.0	89.1	5730	79.3	90.5
1000	3362	2036	61.7	94.8	97.0	6826	94.4	97.4
1500	3466	2142	64.1	97.8	98.7	7059	97.7	98.9
2000	3519	2195	65.3	99.3	99.6	7178	99.3	99.7
2500	3541	2251	66.2	99.9	99.9	7219	99.9	99.9
3000	3545	2316	67.0	100.00	100.0	7228	100.0	100.0
**2008**								
500	476	3684	47.5	54.7	77.3	721	54.4	75.8
1000	685	5331	68.8	78.7	89.3	1050	79.2	89.0
1500	795	6060	78.3	91.4	95.7	1221	92.1	95.8
2000	828	6410	82.7	95.2	97.6	1269	95.7	97.7
2500	843	6598	85.0	96.9	98.4	1289	97.2	98.5
3000	854	6712	86.5	98.2	99.1	1307	98.6	99.2

A positive household refers to a household with an RDT positive resident.

A negative household refers to a household in which all residents are RDT negative.

### Simulated Data from Households Surveyed in 2008

Extrapolation from the households surveyed in 2008 to the non-sampled households resulted in data estimated for 7,961 households with 42,620 individual residents. The household level characteristics of the simulated households did not differ significantly from the sampled households with the exception of the proportion of households with a resident taking antimalarial medication (9.3% in the sampled households vs. 22.1% in the non-sampled households; p = .007) (Table 1). The simulation resulted in 1,340 of 7,961 (16.8%) households with an RDT positive resident, with 470 (35.1%) of these households potentially identified through passive case detection (i.e. index households with a symptomatic, RDT positive individual who would seek care), and 870 (66.7%) households with RDT positive residents likely not to have been identified through passive case detection, either because the infected individual was asymptomatic, lacked care seeking behavior, or both (Table 1).

### Spatial Analysis of Simulated Data from the 2008 Survey Sample

**Figure 4:**
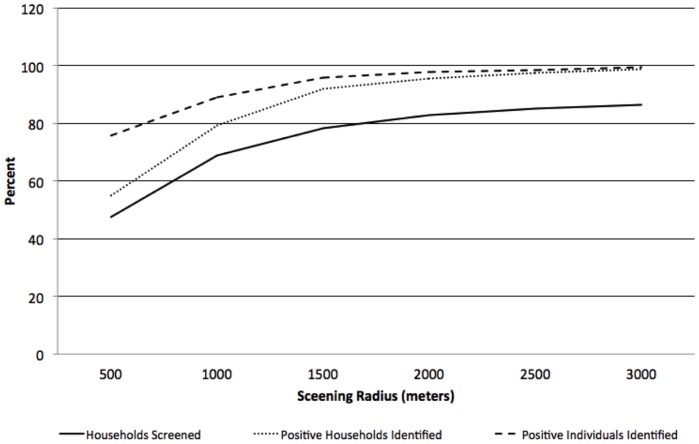
Percentage of RDT positive households identified, RDT positive individuals identified and total households screened by screening radii surrounding index households: 2008.

**Figure 5:**
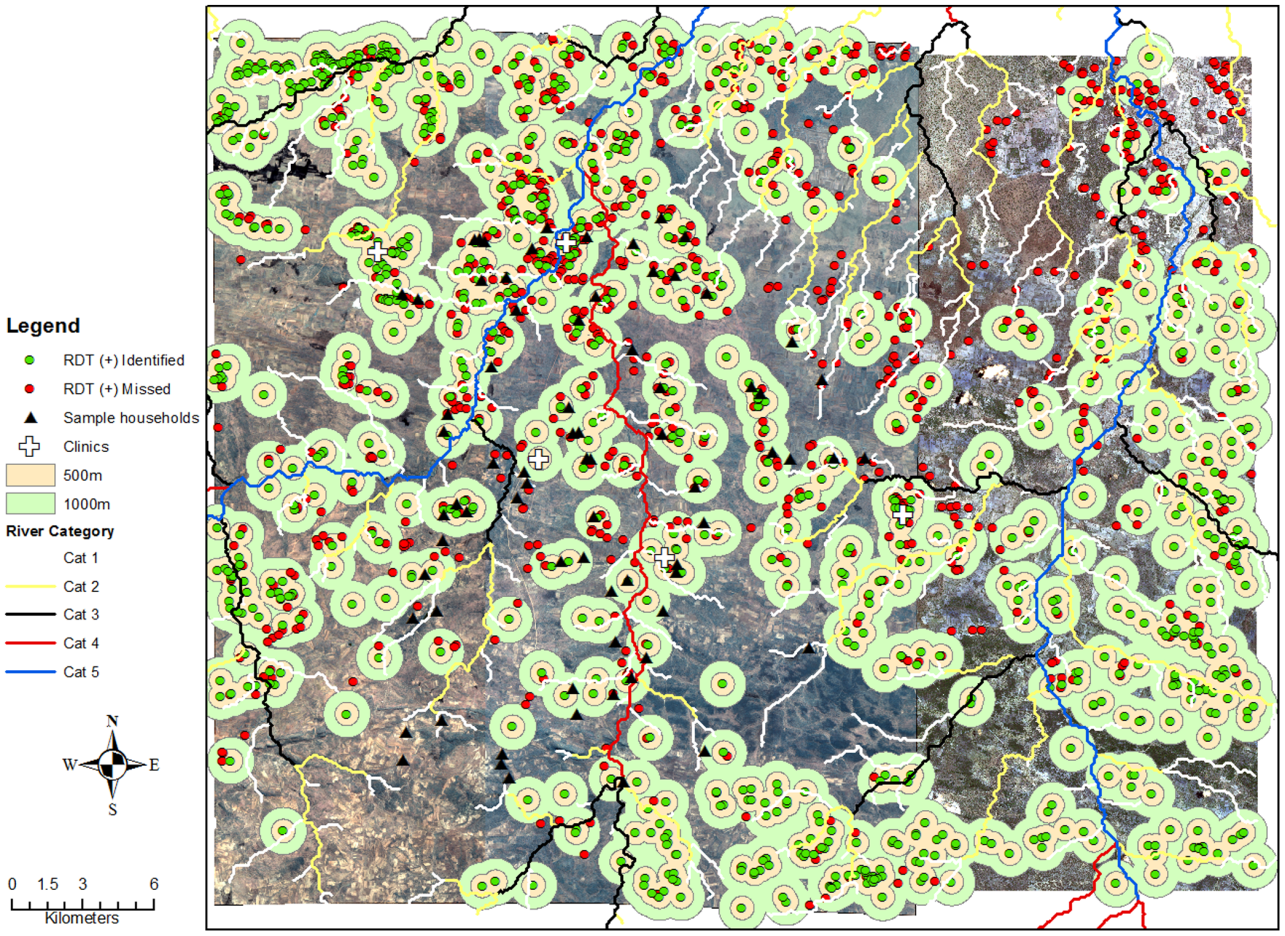
Map of screening radii surrounding RDT positive identified and missed households: 2008.

Of the non-identified households, 476 (54.7%) were located within a 500-meter radius of an index household and 685 (78.7%) were located within a one kilometer radius of an index household (Table 2). When the radius surrounding the index households was expanded to two kilometers, 828 (95.2%) of the positive households were identified and 854 (98.2%) were identified within a radius of 3 kilometers (Table 2, Figure 4). Testing and treating individuals within 500 meters of an index household identified 54.7% of households missed through passive case finding, accounting for over 54.4% of RDT positive individuals who would not have been identified in a health care facility. Of all households within 500 meters, 11% were positive households, with a total of 48% of all households screened (Table 2, Figure 4). When combined with the RDT positive index households and residents, screening all households within 500 meters of an index household would result in identifying 77% of all households with an RDT positive resident and 76% of all RDT positive individuals. If the screening radius was increased from 500 meters to 1 kilometer, combined with the RDT positive index households and residents, 89% of all households with an RDT positive resident would be identified and 89% of all RDT positive individuals, while screening a total of 69% of all households (Table 2, Figure 5).

## Discussion

In areas where malaria transmission has recently declined following implementation of effective control measures, additional strategies are needed to identify and treat infected individuals who do not seek medical care to eliminate gametocyte reservoirs, interrupt transmission and achieve elimination [9]. Extrapolating from data collected in two settings in southern Zambia with different levels of malaria transmission, we demonstrated that reactive case detection within a 500 meter radius from the household of an index case would identify more than three quarters of infected individuals, although the proportion detected was lower as parasite prevalence declined. We are unaware of other published studies that assessed the simulated efficiency of reactive case detection. Testing and treating individuals residing in neighboring households of an index case could be useful in interrupting transmission in regions of declining malaria burden, although cost effectiveness studies are needed to determine the incremental costs associated with expanding the screening radius.

The maps generated by this analysis provide insight into the clustering of RDT positive households under different transmission settings. In addition, the maps show the distances surrounding index households to be screened to maximize the number of infected individuals identified within these foci. In foci where a large proportion of positive households would have been identified by passive case detection, screening and treating household members of an index case would have been sufficient to identify a high proportion of infected individuals. In foci where few households would have been identified passively, screening and treating contacts in the index household and surrounding households would be needed to identify a high proportion of infected individuals.

This analysis showed that reactive case finding has the potential to identify individuals who would have been otherwise missed, simply by screening household members of RDT positive cases that present to the clinic. However, for both transmission settings, the benefits of screening household members was likely insufficient to eliminate the reservoir. Screening within 500 meters of the index households would have a significant impact on identifying and treating a large proportion of the asymptomatic reservoir in both moderate and low transmission settings.

These analyses were based on the results of RDTs to identify infected individuals. However, RDTs are insufficiently sensitive to identify individuals with low-level parasitemia [19], [20], who may account for up to 25% of transmissions to mosquitos [20]. Therefore, our results underestimate the human malaria reservoir. However, reactive case detection as a malaria control and elimination strategy is likely to rely on RDTs for screening, as is currently being done in southern Zambia, and not more sensitive nucleic acid detection tests until low-cost, field friendly assays become available. An alternative strategy to eliminate the infectious reservoir, including those with low-level parasitemia, is to administer ACT and primaquine to all household members of the index case (or within a defined radius) without diagnostic testing. Future analyses may consider the likelihood that undetected infectious individuals become gametocyte carriers and that sufficient mosquitoes feed on them, acquire infection, and become infectious to continue the transmission cycle.

The models were based on several assumptions: the data represent one transmission season; the population was homogeneous with regard to access to care; reinfection did not occur; and complete coverage is achieved of all individuals in all households within the screening radii of identified households shortly after an index case is identified.

The model assumes that these data represent one transmission season; however, the survey sample data was collected cross-sectionally across several months (April–December 2007 and February–December 2008). By making this assumption, any seasonal or temporal trends in malaria incidence were not captured in this analysis. Using data from serial cross-sectional surveys to simulate a closed population without a temporal dimension assumed that spatial clustering of malaria is static and stable over transmission seasons. In support of this assumption, malaria clusters were shown to be fairly stable over time, specifically clusters of asymptomatic parasitemia [21]. However, the spatial clustering of infected individuals is likely seasonal as the force of infection changes, resulting in different efficiencies for reactive case detection within defined radii. Future studies should explore the impact of seasonal malaria transmission on optimal reactive case detection strategies.

The assumption that all persons have equal access to care and treatment may be justified by the multiple health care facilities within the study area and the relatively homogeneous socio-economic status of residents. The assumption regarding reinfection is made likely by data from the longitudinal cohort: 17 of 330 individuals were re-infected in 2007, accounting for 5.2% of the total sample, and only 1 of 435 individuals was re-infected in 2008, accounting for 0.2% of the sample. However, this may be an underestimate due to the effects of repeated treatment within the longitudinal cohort [13]. The potential impact of reactive case detection on onward malaria transmission during this time frame could not be evaluated using this static model. Therefore, the efficiency of reactive case detection in the field may be quite different than the results presented here.

Assumptions were made in extrapolating from sampled to non-sampled households. The simulation was performed based on data from a small but random sample of the entire population. The model fit the data well and was accurate in predicting the data. However, the model was not formally validated externally. While the simulated data did not differ from the sample data, the simulated data may not fully account for heterogeneity between sampled and non-sampled households. The models assumed 100% coverage of all households and residents located within the screening radii of index households. Therefore, the results represent a best-case scenario of the efficiency of reactive case detection. In practice, coverage would not be 100% and the logistics and operational costs, specifically the resources needed to screen all households surrounding index households could impair the feasibility of reactive case detection.

### Conclusions

Identifying and appropriately treating infected individuals, including those who do not seek medical care, is essential to achieve malaria elimination. Reactive case detection may be an additional, important strategy to achieve this goal, although the efficiency may vary in different transmission settings, and cost and effort will likely increase as the transmission level decreases. Testing and treating individuals residing within a defined radius of an index case has the potential to be an effective strategy to identify and treat a large proportion of asymptomatic, minimally symptomatic, and symptomatic individuals who do not seek care in regions with a declining burden of malaria. While this analysis based on the use of RDTs is unable to determine whether reactive case detection can eliminate the human malaria reservoir, including infected individuals who are RDT negative, it can provide insight into the potential impact that may be observed using currently available strategies under different epidemiological conditions.

## Supporting Information

File S1 (DOCX)
