# Supplementary material for: Efficiency of Household Reactive Case Detection for Malaria in Rural Southern Zambia: Simulations Based on Cross-Sectional Surveys from Two Epidemiological Settings
Source: PLoS One. 2013 Aug 6;8(8):e70972. doi: 10.1371/journal.pone.0070972 (PMC3735521; doi:10.1371/journal.pone.0070972)
Supplement: File S1 — (DOCX) [file pone.0070972.s001.docx]

**S1: 2007 Sampled households model fit**

| **Predicted Outcome** | **Method** | **Predictive Covariates** | **AUC** | **p-value goodness of fit** | **R^2^** |
| --- | --- | --- | --- | --- | --- |
| Mean age of household residents | Predictive mean matching | mean age of household residents | NA | NA | NA |
| Number of residents per household | Predictive mean matching | number of residents per household | NA | NA | NA |
| Household RDT status | Logistic regression | Model 1 | .75 | .33 | NA |
| Household antimalarial medication status | Logistic regression | Model 1 | .80 | .45 | NA |
| Number of RDT positive residents per household | Linear regression | Model 2 | NA | NA | .50 |
| Household with a symptomatic resident | Logistic regression | Model 3 | .81 | .67 | NA |
| Household with a care seeking resident | Logistic regression | Model 3 | .84 | .21 | NA |
| RDT positive households with an individual with care seeking behavior and symptoms | Logistic regression | Model 3 | .82 | .78 | NA |
| Antimalarial medication being taken in a household that was received from a care facility | Logistic regression | Model 3 | .78 | .17 |  |

**Model 1** contained the following covariates: mean age of household residents, number of residents per household, ecological risk of the household longitude and latitude.

**Model 2** contained the following covariates: mean age of household residents; number of residents per household, household RDT status, ecological risk of the household, longitude and latitude.

**Model 3** contained the following covariates: mean age of household residents; number of residents per household, household RDT status, number of RDT positive residents per household, household antimalarial medication status, ecological risk of the household, longitude and latitude.

**S2: 2008 Sampled households model fit**

| **Predicted Outcome** | **Method** | **Predictive Covariates** | **AUC** | **p-value goodness of fit** | **R^2^** |
| --- | --- | --- | --- | --- | --- |
| Mean age of household residents | Predictive mean matching | mean age of household residents | NA | NA | NA |
| Number of residents per household | Predictive mean matching | number of residents per household | NA | NA | NA |
| Household RDT status | Logistic regression | Model 1 | .76 | .81 | NA |
| Household antimalarial medication status | Logistic regression | Model 1 | .82 | .96 | NA |
| Number of RDT positive residents per household | Linear regression | Model 2 | NA | NA | .52 |
| Household with a symptomatic resident | Logistic regression | Model 3 | .76 | .76 | NA |
| Household with a care seeking resident | Logistic regression | Model 3 | .70 | .28 | NA |
| RDT positive households with an individual with care seeking behavior and symptoms | Logistic regression | Model 3 | .68 | .27 | NA |
| Antimalarial medication being taken in a household that was received from a care facility | Logistic regression | Model 3 | .83 | .90 | NA |

**Model 1** contained the following covariates: mean age of household residents, number of residents per household, ecological risk of the household longitude and latitude.

**Model 2** contained the following covariates: mean age of household residents; number of residents per household, household RDT status, ecological risk of the household, longitude and latitude.

**Model 3** contained the following covariates: mean age of household residents; number of residents per household, household RDT status, number of RDT positive residents per household, household antimalarial medication status, ecological risk of the household, longitude and latitude.

**S3: 2007 Simulated households model fit**

| **Predicted Outcome** | **Method** | **Predictive Covariates** | **AUC** | **p-value goodness of fit** | **R^2^** |
| --- | --- | --- | --- | --- | --- |
| Mean age of household residents | Predictive mean matching | mean age of household residents | NA | NA | NA |
| Number of residents per household | Predictive mean matching | number of residents per household | NA | NA | NA |
| Household RDT status | Logistic regression | Model 1 | .88 | .18 | NA |
| Household antimalarial medication status | Logistic regression | Model 1 | .93 | .07 | NA |
| Number of RDT positive residents per household | Linear regression | Model 2 | NA | NA | .40 |
| Household with a symptomatic resident | Logistic regression | Model 3 | .85 | .35 | NA |
| Household with a care seeking resident | Logistic regression | Model 3 | .94 | .21 | NA |
| RDT positive households with an individual with care seeking behavior and symptoms | Logistic regression | Model 3 | .85 | .22 | NA |
| Antimalarial medication being taken in a household that was received from a care facility | Logistic regression | Model 3 | .86 | .33 | NA |

**Model 1** contained the following covariates: mean age of household residents, number of residents per household, ecological risk of the household longitude and latitude.

**Model 2** contained the following covariates: mean age of household residents; number of residents per household, household RDT status, ecological risk of the household, longitude and latitude.

**Model 3** contained the following covariates: mean age of household residents; number of residents per household, household RDT status, number of RDT positive residents per household, household antimalarial medication status, ecological risk of the household, longitude and latitude.

**S4: 2008 Simulated households model fit**

| **Predicted Outcome** | **Method** | **Predictive Covariates** | **AUC** | **p-value goodness of fit** | **R^2^** |
| --- | --- | --- | --- | --- | --- |
| Mean age of household residents | Predictive mean matching | mean age of household residents | NA | NA | NA |
| Number of residents per household | Predictive mean matching | number of residents per household | NA | NA | NA |
| Household RDT status | Logistic regression | Model 1 | .80 | .76 | NA |
| Household antimalarial medication status | Logistic regression | Model 1 | .80 | .30 | NA |
| Number of RDT positive residents per household | Linear regression | Model 2 | NA | NA | .78 |
| Household with a symptomatic resident | Logistic regression | Model 3 | .88 | .89 | NA |
| Household with a care seeking resident | Logistic regression | Model 3 | .72 | .29 | NA |
| RDT positive households with an individual with care seeking behavior and symptoms | Logistic regression | Model 3 | .82 | .43 | NA |
| Antimalarial medication being taken in a household that was received from a care facility | Logistic regression | Model 3 | .87 | .20 | NA |

**Model 1** contained the following covariates: mean age of household residents, number of residents per household, ecological risk of the household longitude and latitude.

**Model 2** contained the following covariates: mean age of household residents; number of residents per household, household RDT status, ecological risk of the household, longitude and latitude.

**Model 3** contained the following covariates: mean age of household residents; number of residents per household, household RDT status, number of RDT positive residents per household, household antimalarial medication status, ecological risk of the household, longitude and latitude.
